# Risk factors and predictive nomograms for bedside emergency endoscopic treatment following endotracheal intubation in cirrhotic patients with esophagogastric variceal bleeding

**DOI:** 10.1038/s41598-024-59802-0

**Published:** 2024-04-24

**Authors:** Ajuan Zeng, Yangjie Li, Lingna Lyu, Shibin Zhang, Yuening Zhang, Huiguo Ding, Lei Li

**Affiliations:** 1 Department of Gastroenterology and Hepatology, Beijing You’an Hospital Affiliated to Capital Medical University, Beijing, 100069 China; 2 Department of Gastroenterology, Beijing Jishuitan Hospital, Capital Medical University, Beijing, 102208 China

**Keywords:** Gastroenterology, Risk factors

## Abstract

Data on emergency endoscopic treatment following endotracheal intubation in patients with esophagogastric variceal bleeding (EGVB) remain limited. This retrospective study aimed to explore the efficacy and risk factors of bedside emergency endoscopic treatment following endotracheal intubation in severe EGVB patients admitted in Intensive Care Unit. A total of 165 EGVB patients were enrolled and allocated to training and validation sets in a randomly stratified manner. Univariate and multivariate logistic regression analyses were used to identify independent risk factors to construct nomograms for predicting the prognosis related to endoscopic hemostasis failure rate and 6-week mortality. In result, white blood cell counts (*p* = 0.03), Child-Turcotte-Pugh (CTP) score (*p* = 0.001) and comorbid shock (*p* = 0.005) were selected as independent clinical predictors of endoscopic hemostasis failure. High CTP score (*p* = 0.003) and the presence of gastric varices (*p* = 0.009) were related to early rebleeding after emergency endoscopic treatment. Furthermore, the 6-week mortality was significantly associated with MELD scores (*p* = 0.002), the presence of hepatic encephalopathy (*p* = 0.045) and postoperative rebleeding (*p* < 0.001). Finally, we developed practical nomograms to discern the risk of the emergency endoscopic hemostasis failure and 6-week mortality for EGVB patients. In conclusion, our study may help identify severe EGVB patients with higher hemostasis failure rate or 6-week mortality for earlier implementation of salvage treatments.

## Introduction

Esophagogastric variceal bleeding (EGVB) is one of the aggressive complications of portal hypertension in cirrhosis with high mortality rates^1,2^. The management and treatment of EVGB involves the coordination of multidisciplinary departments, focusing on emergency hemostasis, hemodynamic resuscitation, airway protection, pharmacologic therapy, and endoscopic or interventional radiology therapy. Of them, endoscopic procedures remain the mainstay recommended by domestic and international guidelines for hemorrhagic control of EGVB^3–5^. Despite the marked advances in the management of portal hypertension and EGVB in recent years, the failure rate of hemostasis treatment remains as high as 10–20%, leading to a notable increase in and the risk of mortality^6–8^, while severe EGVB patients may have higher mortality. Therefore, assessing the risk factors associated with emergency endoscopic hemostasis treatment and early rebleeding are necessary to determine the clinical prognosis of EGVB patients.

Patients with severe EGVB with significant mortality are often admitted to ICU for emergency endoscopic treatment under airway protection and organ support. Generally, When EGVB patients is accompanied by massive bleeding, unconsciousness or agitation, there is a high risk of aspiration and hemorrhagic shock during acute episodes. Intubation and emergency bleeding control are the two essential steps for improving oxygenation and reduction the incidence of malabsorption to enhance the effectiveness and safety of endoscopic treatment. Therefore, it is imperative to maintain the airway integrity by intubation in cirrhotic patients with impaired consciousness and those actively vomiting blood^5^. Most guidelines suggest that intubation is recommended in EGVB patients before emergency endoscopy therapy^9,10^. In contrast, some studies have found that performing endoscopic procedures under mechanically assisted ventilation poses risks such as decannulation, asphyxia, aspiration and respiratory cardiac arrest. Compared with endoscopic treatment without tracheal intubation, endoscopic treatment with tracheal intubation is associated with a higher incidence of pneumonia, prolonged hospitalization, mortality and increased hospital costs in patients with EGVB^11^. In together, assessing various risk factors and risk stratification of bedside emergency endoscopic treatment in severe EGVB patients following endotracheal intubation is important in effort to identify the group of patients at high risk of hemostasis failure and mortality.

This study retrospectively analyzed and compared the clinical data of emergency bedside endoscopic treatment of patients with severe EGVB in ICU. Our main objective was to quantify the clinical efficacy and risk factors of emergency bedside endoscopic treatment following the tracheal intubation in severe EGVB patients admitted to ICU, and to establish nomogram models to predict its associated risks and prognosis.

## Methods

### Patients and data acquisition

Cirrhotic patients who were diagnosed with acute upper gastrointestinal bleeding through bedside emergency endoscopic treatment at Beijing You’an Hospital affiliated to Capital Medical University between June 2016 and June 2023 were retrospectively retrieved in the present study. Two researchers independently included patients according to the study inclusion criteria and exclusion criteria to avoid selection bias. The inclusion criteria were as follows: (1) the diagnosis of EGVB was based on one or more of the following signs on endoscopy: (i) active bleeding or blood oozing of the varicose veins; (ii) no obvious bleeding foci but several signs of recent variceal bleeding (stain), such as the formation white thrombus thrombotic head or an overlying blood clot; and (2) acute severe EGVB patients with shock, actively vomiting blood, unconsciousness or agitation who underwent bedside emergency endoscopic treatment under endotracheal intubation protection in ICU. Exclusion criteria were as follows: (1) age < 18 or > 80 years; (2) non-varicose bleeding or the cause of bleeding undetermined; (3) complications with malignancy other than liver cancer. The general clinical characteristics and laboratory values, including demographic information (age, sex, smoking, drinking), complications (ascites, hepatic encephalopathy (HE), portal vein thrombosis (PVT), liver failure, etc.), relevant laboratory parameters (white blood cell (WBC) counts, hemoglobin (Hb) and platelet counts (PLT), alanine aminotransferase (ALT), aspartate aminotransferase (AST), total bilirubin (TBIL), albumin concentrations (ALB), Cholinesterase (CHE), serum creatinine(Cr), eGFR, prothrombin activity (PTA), international normalized ratio (INR) and other clinical features (the location of bleeding and treatment modality) were collected through the electronic medical record system. The degree of ascites was classified as none, mild, or moderate- severe according to International Ascites Club. HE was classified as none, latent, overt according to EASL (European Association for the Study of the Liver) Clinical Practice Guidelines^12^. Child-Turcotte-Pugh (CTP) scores and Model for End-Stage Liver Disease (MELD) scores were to evaluate the severity of liver disease in each patient^13^. Notably, the values of MELD scores have ranged from 6 to 40 (with values above 40 being treated equally to 40) since 2002. However, the number of recipients with MELD scores over 40 increasing 4.8 times associated higher mortality over the past 20 years. MELD scores beyond 40 are related to increase waitlist mortality without adversely affecting post-transplant outcome, suggesting that uncapping the MELD score could increase survival benefits. Therefore, our study uncapped the MELD score and retained the original scores.

### Endoscopic treatment procedures

All bedside emergency endoscopic treatments in the ICU were performed by senior gastroenterologists who were experienced in the management of esophageal and gastric varices. All patients underwent emergency endoscopic treatment according to the location and degree of the varices: endoscopic variceal ligation (EVL), endoscopic injection sclerotherapy (EIS), and endoscopic tissue adhesive (ETA) within 12–24 h of onset after haemodynamic resuscitation. Antibiotics is were instituted from admission. After the operation, portal pressure reducing drugs, including somatostatin and octreotide, were administered over a course of 4 days at a rate of 250–500 µg/h and 25–50 µg/h, respectively. Proton pump inhibitors were used for 2–4 weeks. The vital signs of patients were closely monitored and the patients were gradually transitioned to a normal diet.

### Outcomes measurements

Primary outcome was the hemostasis failure rate after emergency endoscopic treatment. Hemostasis failure was defined as the reappearance of signs of upper gastrointestinal bleeding within 72 h after the initial endoscopic hemostasis, such as hematemesis, hematochezia, falling hemoglobin (hemoglobin drops > 30 g/L without blood transfusion) and hemorrhagic shock. Secondary endpoints included: (1) early rebleeding: active bleeding within 72 h to 6 weeks after the first bleeding was controlled; (2) 6-week mortality.

### Statistical analysis

All statistical analyses were conducted by using SPSS version 22.0 (IBM, SPSS Statistics, Armonk, NY) and R version 4.2.1 (The R Foundation for Statistical Computing, Vienna, Austria). All enrolled patients were randomly divided into a training set and a validation set in a ratio of 2:1. Descriptive statistics were calculated for Descriptive all demographic and clinical characteristics of patients were calculated. Shapiro Wilk test was used to test the normality of quantitative data. Data were represented as the mean ± Standard Error of Mean (SEM), or as median with interquartile ranges (IQR). Categorical variables were represented as the number of cases and percentages. Univariate analysis was performed by using t test for continuous variables, and the chi-square test and the Fisher's precision probability test for categorical variables. The variables for inclusion in the multivariate binary logistic regression analysis were chosen based on univariate analysis with a cutoff *p* value of 0.10 to calculate the and odds ratio (OR) value to identify the independent risk factors. The statistics were all bilateral tests, and *p* < 0.05 was considered as significantly statistical difference.

Nomogram models were established based on the multivariable logistic regression analysis results and validated using bootstrap resampling. The predictive effectiveness and discrimination performance of the nomogram was evaluated by the area under the curve (AUC) and calibration (using Hosmer–Lemeshow test, *p* > 0.05 indicating no significant differences between expected and observed outcomes)^14^. The cumulative survival rates were estimated by the Kaplan–Meier method and comparisons between the curves were made with the log-rank test.

### Ethical approval and consent to participate

This study was approved and consented to by the Ethics Committee of Beijing You’an Hospital in accordance with the Declaration of Helsinki. Informed consent was obtained from all subjects or their legal guardians and the data used were anonymous.

## Results

### Clinical characteristics of EGVB patients who receiving emergency endoscopic treatment with endotracheal intubation

**Figure 1:**
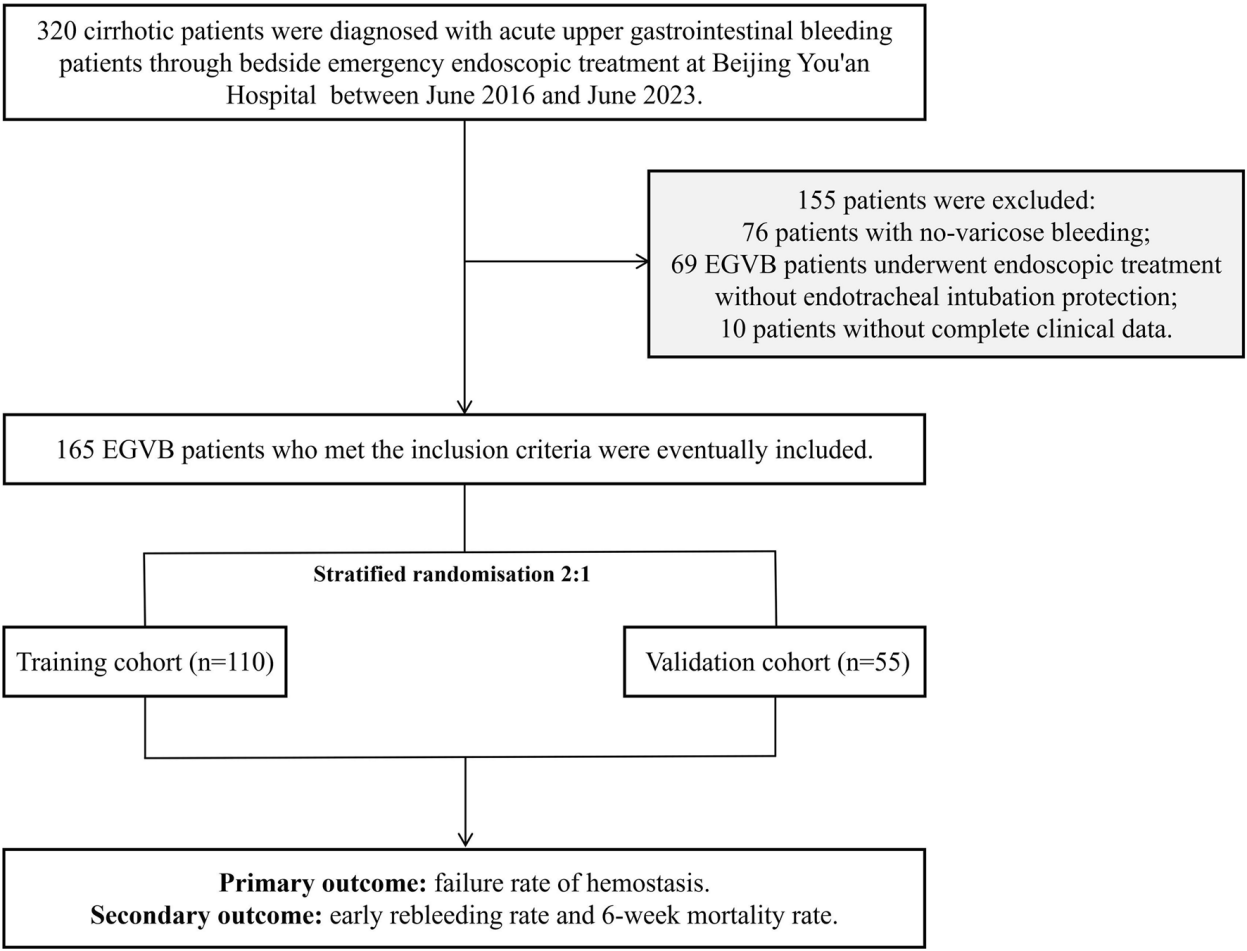
Flow diagram of the study.

According to the inclusion and exclusion criteria, a total of 165 EGVB patients who underwent bedside emergency endoscopic treatment with endotracheal intubation protection in the ICU were included in this study (Fig. 1). Among these, 125 were males (75.8%), and the mean age, WBC, PLT, ALB, and PTA were 56.5 ± 0.9 (years), 88.4 ± 4.3 (10^9^/L), 26.7 ± 0.5 (g/L) and 46.2 ± 1.5 (%), respectively. The median (IQR) levels of CTP scores and MELD scores were 11 (IQR 9–13) and 14 (IQR 10–21) respectively. The distribution of hepatic encephalopathy was as follows: None, 69 (41.8%) patients; I-II, 44 (26.7%) patients; and III-IV, 52 (31.5%) patients. All patients were randomly divided into training and validation sets in a 2:1 ratio. The clinical characteristics and examination indicator distributions were comparable between the training and validation sets (*p* > 0.05, Table 1).

**Table 1:** Baseline characteristics of patients with EGVB in the training and validation set.

Variables	Total (n = 165)	Training set (n = 110)	Validation set (n = 55)	*P* value
Age	56.5 ± 0.9	55.6 ± 1.1	56.2 ± 1.6	0.97
WBC (10^9^/L)	9.0 ± 0.4	8.8 ± 0.5	9.3 ± 0.9	0.82
Hb (g/L)	61.00 (50–72.3)	63.00 (52–71)	61 (50–71.5)	0.97
PLT (10^9^/L)	88.4 ± 4.3	85.1 ± 5.1	94.9 ± 7.6	0.55
ALT (U/L)	25 (14–51)	26 (16–54)	23 (12–51)	0.66
AST (U/L)	43 (24–108.5)	43.5 (24–148)	41 (23–94)	0.81
TBIL (μmol/L)	34.2 (20.6–31)	39.7 (23.05–116.7)	30.1 (18.2–52.4)	0.1
ALB (g/L)	26.7 ± 0.5	26.5 ± 0.6	27.1 ± 0.8	0.84
PT (s)	19.5 (15.7–25.7)	20 (15.8–27.1)	18.3 (15.6–22.5)	0.37
PTA (%)	46.2 ± 1.5	44.3 ± 1.7	50.1 ± 2.6	0.17
INR	1.7 (1.4–2.2)	1.8 (1.4–2.3)	1.63 (1.4–2)	0.29
CTP scores	11 (9–13)	11 (9–13)	11 (8–13)	0.57
MELD scores	14 (10–21)	15 (11.8–23.3)	12 (10–18)	0.14
Sex, male	125 (75.8)	84 (76.4)	41 (74.5)	0.97
Bleeding site, EV	123 (75.2)	82 (75.5)	41 (74.5)	0.99
Blakemore tube	73 (44.2)	46 (41.8)	27 (49.1)	0.68
Treatment	57 (34.5)	43 (39.1)	14 (25.5)	0.225
TAI/EIS/EVL	79 (47.9)	58 (52.7)	21 (38.2)	0.21
TAI + EIS/EVL	86 (52.1)	52 (47.3)	34 (61.8)	
Shock	99 (60)	69 (62.7)	30 (54.5)	0.6
First bleeding	70 (42.4)	45 (40.9)	25 (45.5)	0.86
Portal vein emboli				
Liver cancer	27 (22.4)	29 (26.4)	8 (14.5)	0.23
Hepatic failure	65 (39.4)	45 (40.9)	20 (36.4)	0.85
HE				0.99
None	69 (41.8)	47 (42.7)	22 (40.0)	
Latent	44 (26.7)	28 (25.5)	16 (29.1)	
Overt	52 (31.5)	35 (31.8)	17 (30.9)	
Ascites				0.49
None	11 (6.7)	6 (5.5)	5 (9.1)	
Mild	72 (43.6)	44 (40.0)	28 (50.9)	
Moderate-severe	82 (49.7)	60 (54.5)	22 (40.0)	
Treatment timing				0.99
< 12 h	128 (77.6)	85 (77.3)	43 (78.2)	
> 12 h	37 (22.4)	25 (22.7)	12 (21.8)	

### Analysis of predictive risk factors in the failure rate of emergency endoscopic hemostasis

Based on the presence or absence of active bleeding within 72 h after emergency endoscopic treatment, patients were divided into two groups: the successful hemostasis group and the failed hemostasis group. The failure rate of endoscopic hemostasis was 32.12% (53/165). In the training cohort, univariate logistic regression analyses identified that higher WBC counts, TBIL, ALT, AST, eGFR, PT, PTA, INR, CPT score, MELD score, comorbid shock, hepatic failure, HE and ascites were significantly associated with the failure of endoscopic hemostasis. Multivariate logistic regression analyses showed that WBC counts (OR 1.1, 95% Confidence Interval (CI) 1.01–1.2; *P* = 0.030), CTP score (OR 1.4; 95% CI 1.1–1.7; *P* = 0.001) and shock (OR 4.5, 95% CI 1.5–13.7; *P* = 0.005) were independently associated with endoscopic hemostasis failure (Table 2).

**Table 2:** Risk factors of endoscopic hemostasis failure in EGVB patients in training cohort.

Variables	emergency endoscopic hemostasis		Univariate		Multivariate	
	success (n = 75)	failure (n = 35)	OR (95% CI)	*p* value	OR (95% CI)	*P* value
Age	56.3 ± 1.5	57.3 ± 1.6	1 (0.5–2.3)	0.65		
WBC (10^9^/L)	6.61 (3.8–9.8)	10.6 (7.3–13.2)	2.3 (1.6–3.5)	< 0.001	1.1 (1–1.2)	0.03
Hb (g/L)	61 (48–71)	64 (51—82)	1.3 (0.8–2.9)	0.506		
PLT (10^9^/L)	83.1 ± 6.2	89.3 ± 9.3	1.1 (0.5–2.5)	0.579		
ALT (U/L)	23 (12–46)	44 (23–67)	2.4 (1.1–5.6)	0.006	0.99 (0.99–1)	0.588
AST (U/L)	38 (22–97)	83 (37–235)	0.7 (0.3–1.6)	0.002	1 (1–1.01)	0.057
TBIL (μmol/L)	31.9 (17.8–79.2)	75.9 (32.2–185.8)	1.5 (1.1–2.1)	0.003	1 (0.99–1)	0.522
ALB (g/L)	27.1 ± 0.7	25.12 ± 1.11	0.5 (0.2–1.2)	0.104		
PT (s)	18.7 (15–24.3)	24.1 (18.2–33.1)	2.6 (1.1–6.1)	0.002	0.9 (0.8–1.1)	0.349
PTA (%)	47.7 ± 2.1	37.0 ± 2.8	0.6 (0.3–1)	0.004	1 (0.98–1.1)	0.392
INR	1.7 (1.4–2.1)	2.1 (1.64–2.9)	3.5 (1.4–8.4)	0.001	1.3 (0.6–2.6)	0.408
CTP scores	10 (8–12)	13 (11–14)	1.7 (1.3–2.3)	< 0.001	1.4 (1.1–1.7)	0.001
MELD scores	14 (10–20)	19 (14–28)	3.2 (1.3–7.6)	0.004	1 (0.9–1)	0.268
Sex, male	55 (73.3)	29 (82.9)	1.8 (0.7–4.9)	0.273		
Bleeding site, EV	56 (76)	26 (74.3)	1 (0.5–2.0)	0.996		
Blakemore tube	31 (41.3)	15 (42.9)	1.1 (0.5—2.4)	0.88		
Treatment			1.5 (0.7–3.5)	0.297		
TAI/EIS/EVL	37 (49.3)	21 (60)				
TAI + EIS/EVL	38 (50.7)	14 (40)				
Shock	39 (52)	30 (85.7)	5.5 (1.9–15.8)	0.001	4.5 (1.5–13.7)	0.005
First bleeding	31 (41.3)	14 (40)	1 (0.4–2.1)	0.895		
Portal vein emboli	32 (42.7)	11 (31.4)	0.6 (0.3–1.4)	0.262		
Liver cancer	19 (25.3)	10 (28.6)	1.2 (0.5–2.9)	0.72		
Hepatic failure	23 (30.7)	22 (62.9)	2.8 (1.7–8.9)	0.001	3.2 (0.5–19.8)	0.718
HE				< 0.001		0.139
None	37 (49.3)	10 (28.6)	Reference			
Latent	23 (30.7)	5 (14.3)	0.8 (0.3–2.2)	0.722	0.4 (0.1–1.6)	0.072
Overt	15 (20)	20 (57.1)	4.9 (1.9–13)	0.001	1.4 (1–2.0)	0.09
Ascites				0.056		0.689
None	6 (8)	0 (0)	Reference			
Mild	33 (44)	11 (31.4)	3.6 (0.2–54.2)	0.358	> 100 (0–Inf)	0.579
Moderate-severe	36 (48)	24 (68.6)	5.6 (0.4–82.7)	0.208	> 100 (0–Inf)	0.478
Treatment timing			0.6 (0.3–1.6)	0.99		
< 12 h	60 (80)	25 (71.4)				
> 12 h	15 (20)	10 (28.6)				

**Figure 2:**
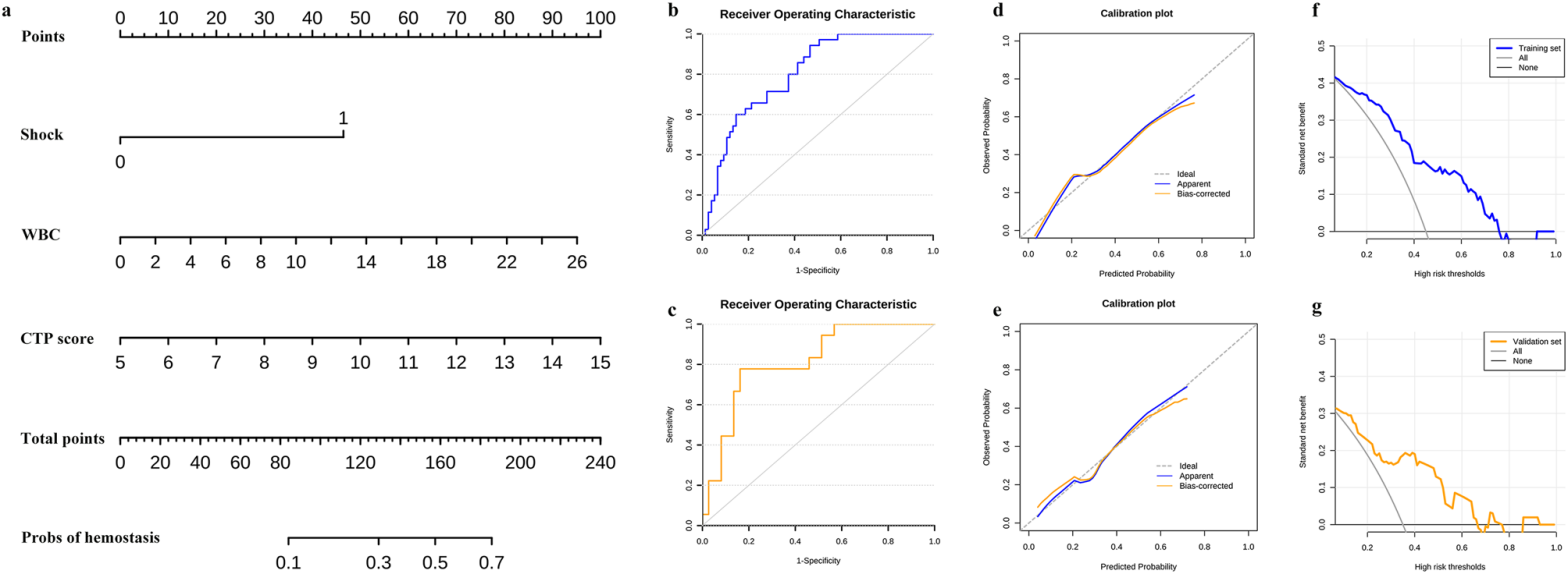
The nomogram for predicting the failure rate of emergency endoscopic hemostasis in acute EGVB patients. (**a**) The total score and the density map of shock (0 representing patients without shock, 1 representing comorbid shock) WBC, and CPT score are shown. Each category of the variables is ordered according to the standard deviation on the nomogram scale. Draw a line down to the probability axis to identify the risk of hemostasis failure. (**b**) and (**c**) were shown the ROC curves of the nomogram in training and validation set respectively. (**d**) and (**e**) were shown the calibration curves in training and validation set respectively. (**f**) and (**g**) were shown the decision curves for prediction of the net benefit of the constructed nomogram in training and validation set.

A new predictive nomogram for the emergency endoscopic hemostasis failure in patients with EGVB was constructed based on the corresponding independent risk factors (Fig. 2a). The AUC and calibration curves were used to evaluate the efficacy of the predictive model. The AUC the nomogram used to predict the rate of emergency endoscopic hemostasis failure was 0.801 (95% CI 0.72–0.89) in the training set (Fig. 2b) and 0.82 (95% CI 0.69–0.93) in the validation set (Fig. 2c) to predict the rate emergency endoscopic hemostasis failure. Calibration plot describes the consistency of nomogram scores between the expected and observed rates of hemostasis failure. The apparent curve (actual) and bias-corrected curve (500 times bootstrapped adjusted) were all very close to the ideal curve which showed good agreement in both the training set (Fig. 2d , *p* = 0.574) and validation set (Fig. 2e , *p* = 0.532), indicating that the model fits well. Next, the decision curve analysis (DCA) curve was employed to evaluate the predictive performance. The results demonstrated that the threshold probability range was 4–75% (Fig. 2f) in the training set and 4–92% (Fig. 2g) in the validation set for the nomogram’s ability to predict the failure rate of hemostasis. In summary, the risk prediction model for endoscopic hemostasis failure indicated a broad range of threshold probabilities with good accuracy.

### Risk factors of early rebleeding after bedside emergency endoscopic treatment

To investigate the risk factors for early rebleeding (defined as the recurrence of bleeding within 72 h to 6 weeks) after emergency endoscopic treatment. EGVB patients with successful hemostasis in the training set were further divided into early rebleeding and non-early rebleeding groups. Early rebleeding occurred in 22 of the 75 patients with successful hemostasis, with an overall rebleeding rate of 29.3%. CTP score, bleeding location and the presence of hepatic encephalopathy were found to be related to the risk of early rebleeding. Multivariate logistic regression analyses showed that high CTP score (OR 1.5, 95% CI 1.2–1.9; *p* = 0.003) and gastric varices (OR 5.9, 95% CI 1.6–22.1; *p* = 0.009) were independent predictors of early bleeding (Table S1).

### Risk factors analysis and construction a predictive nomogram of 6-week mortality

A total of 75 EGVB patients (75/165, 45.45%) died within the 6-week follow-up period after bedside emergency endoscopic hemostasis accompanied with endotracheal intubation. To analyze the risk factors associated with 6-week mortality after emergency endoscopic treatment in EGVB patients, the 110 EGVB patients from the training set were divided into the death group (n = 51) and non-death group (n = 59) according to follow-up data. In the training cohort, the univariate logistic regression analysis indicated that WBC, ALT, AST, TBIL, PT, PTA, INR, CTP scores, MELD scores, treatment modality, shock, hepatic failure, hepatic encephalopathy, ascites and postoperative rebleeding were significantly associated with 6-week mortality in active EGVB patient who underwent emergency endoscopic treatment with endotracheal intubation. Multivariate logistic regression analysis results revealed that high MELD scores (OR 1.2, 95% CI 1.1–1.3, *p* = 0.002), the presence of hepatic encephalopathy (*p* = 0.045) and postoperative rebleeding (*p* < 0.001) were independent risk factors for the composite outcomes in 6-week mortality by multivariate analysis (Table 3).

**Table 3:** The predictors of 6-week mortality in EGVB patients in training cohort.

Variables	Death		Univariate		Multivariate	
	Yes (n = 51)	No (n = 59)	OR (95%CI)	*p* value	OR (95%CI)	*p* value
Age	56.8 ± 1.2	56.4 ± 1.8	0.6 (0.3–1.3)	0.867		
WBC (109/L)	9.7 (6.7–12.7)	5.8 (3.5–9.8)	3.5 (1.6–7.8)	0.001	1 (0.9–1.2)	0.994
Hb (g/L)	61 (51–75)	61.00 (48–71)	1 (0.5–2.2)	0.945		
PLT (10^9^/L)	87.3 ± 8.1	83.2 ± 6.6	1 (0.5–2.2)	0.69		
ALT (U/L)	40 (23–67)	20 (12–38)	3.9 (1.8–8.2)	< 0.001	1 (1–1.01)	0.934
AST (U/L)	89 (39–231)	32 (20,60)	4.3 (1.9–9.5)	< 0.001	1 (0.99–1)	0.268
TBIL (μmol/L)	75.9 (31–213.4)	28.5 (17.4–50.1)	5.5 (2.4–12.4)	< 0.001	1 (0.99–1.01)	0.55
ALB (g/L)	25.5 ± 0.9	27.3 ± 0.7	0.5 (0.2–1)	0.121		
PT (s)	25.1 (19.9–33.4)	17.3 (14.8–22.1)	5.1 (2.2–11.4)	< 0.001	0.9 (0.7–1.9)	0.328
PTA (%)	35.93 ± 2.5	50.7 ± 2.1	0.2 (0.1–0.4)	< 0.001	1.1 (0.98–1.1)	0.165
INR	2.2 (1.8–3)	1.6 (1.3–2)	7.2 (3.1–16.8)	< 0.001	1.4 (0.5–3.7)	0.499
CTP scores	13 (11–14)	10 (8–12)	6.5 (2.8–15)	< 0.001	1 (0.7–1.6)	0.895
MELD scores	20 (14–30)	13 (9–16)	5.9 (2.6–13.5)	< 0.001	1.2 (1.1–1.3)	0.002
Sex	43 (84.3)	41 (69.5)	0.4 (0.2–1.1)	0.068	1.4 (0.2–9.1)	0.716
Bleeding site, EV	39 (76.5)	43 (72.9)	0.8 (0.4–2)	0.667		
Blakemore tube	21 (41.2)	25 (42.4)	1.1 (0.5–2.3)	0.889		
Treatment			1.4 (1–2)	0.052	0.3 (0.1–1.4)	0.123
TAI/EIS/EVL	32 (62.7)	26 (44.1)				
TAI + EIS/EVL	19 (37.3)	33 (55.9)				
Shock	37 (72.5)	32 (54.2)	2.2 (1–5)	0.048	2.4 (0.5–12.2)	0.276
First bleeding	23 (45.1)	22 (37.3)	0.7 (0.3–1.6)	0.406		
Portal vein emboli	17 (33.3)	26 (44.1)	1.6 (0.7–3.4)	0.25		
Liver cancer	16 (31.4)	13 (22)	0.6 (0.3–1.5)	0.268		
Hepatic failure	32 (62.7)	13 (22)	6 (2.6–13.8)	< 0.001		
HE				< 0.001		0.045
None	13 (25.5)	34 (57.6)	Reference		Reference	
Latent	8 (15.7)	20 (33.9)	1.05 (0.4–3)	0.932	0.5 (0.1–2.3)	0.405
Overt	30 (58.8)	5 (8.5)	15.7 (5–49.2)	< 0.001	4.8 (1–23.1)	0.052
Ascites				< 0.001		0.369
None	0 (0)	6 (10.2)	Reference			
Mild	13 (25.5)	31 (52.5)	5.6 (0.3–106)	0.253	> 100 (0–Inf)	0.999
Moderate-severe	38 (74.5)	22 (37.3)	22.2 (1.2–413)	0.038	> 100 (0–Inf)	0.999
Treatment timing			2 (0.8–5.1)	0.012		
< 12 h	36 (70.6)	49 (83.1)				
> 12 h	15 (29.4)	10 (16.9)				
Rebleeding				< 0.001		< 0.001
None	7 (13.7)	46 (78.0)	Reference		Reference	
72 h	31 (60.8)	4 (6.78)	50.9 (13.7–188)	< 0.001	52.23 (10.3–265)	< 0.001
72 h–6 weeks	13 (25.5)	9 (15.2)	9.5 (2.1–9.7)	< 0.001	17.1 (3.3–88.2)	0.001

**Figure 3:**
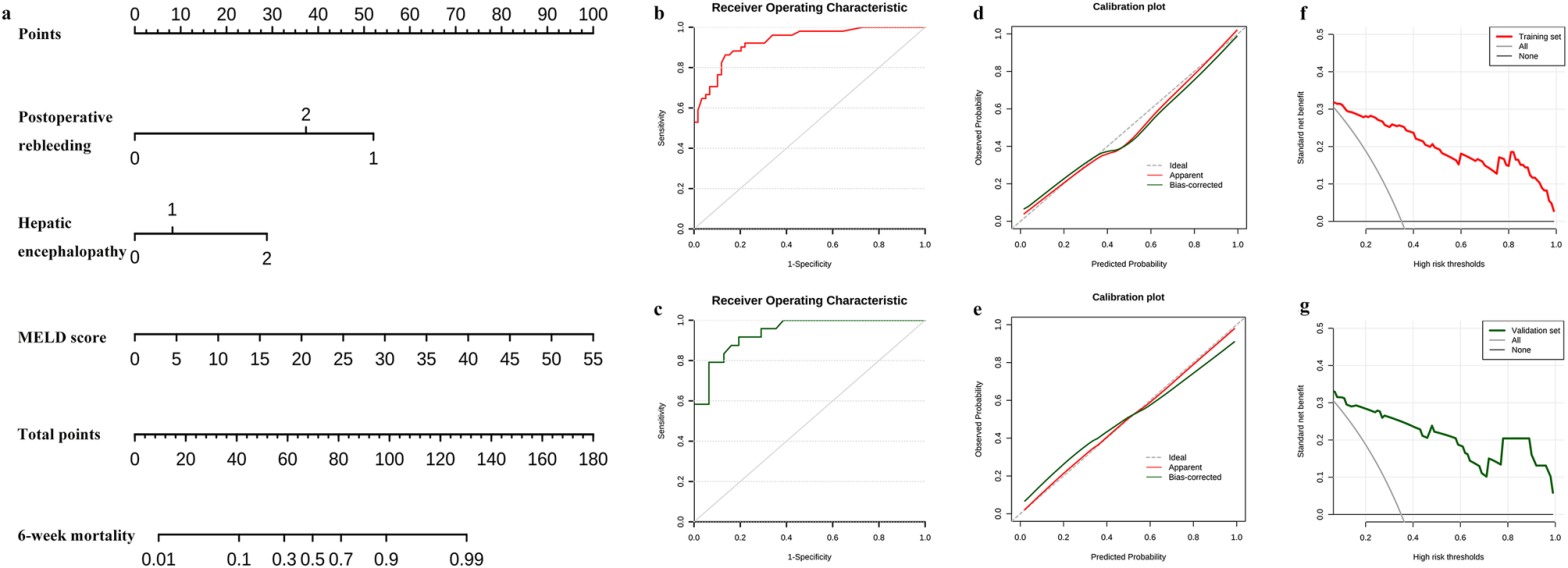
The nomogram for predicting the 6-week mortality in acute EGVB patients who received bedside emergency endoscopic treatment following endotracheal intubation. (**a**) The total score and the density map of postoperative rebleeding (0 representing patients without rebleeding, 1 representing rebleeding in 72 h, 2 representing rebleeding in 72 h–6 weeks), hepatic encephalopathy (0 representing patients without HE, 1 representing latent HE, 2 representing overt HE) and MELD scores. The score of each variable is assigned a score on the Points scale. The sum of these scores is located on the Total points scale and a line is drawn downward to determine the specific 6-week mortality. (**b**) and (**c**) were shown the ROC curves of the nomogram in training and validation set respectively. (**d**) and (**e**) were shown the calibration curves in training and validation set respectively. (**f**) and (**g**) were shown the decision curves in training and validation set.

All significant predictors were incorporated into a nomogram to predict the risk of 6-week mortality (Fig. 3a). The AUCs of the nomogram were 0.93 (95% CI 0.88–0.98), and 0.94 (95% CI 0.88–1) in the training (Fig. 3b) and validation (Fig. 3c) set, respectively. Furthermore, the calibration curves in both the training (*p* = 0.593, Fig. 3d) and validation cohorts (*p* = 0.844, Fig. 3e) showed close agreement between the real and projected results by the column line plots. The DCA showed good predictive efficiency in the column line graphs for 6-week mortality in EGVB patients who received emergency endoscopic treatment with endotracheal intubation with a 1–99% probability range both in the training (Fig. 3f) and validation set (Fig. 3g).

**Figure 4:**
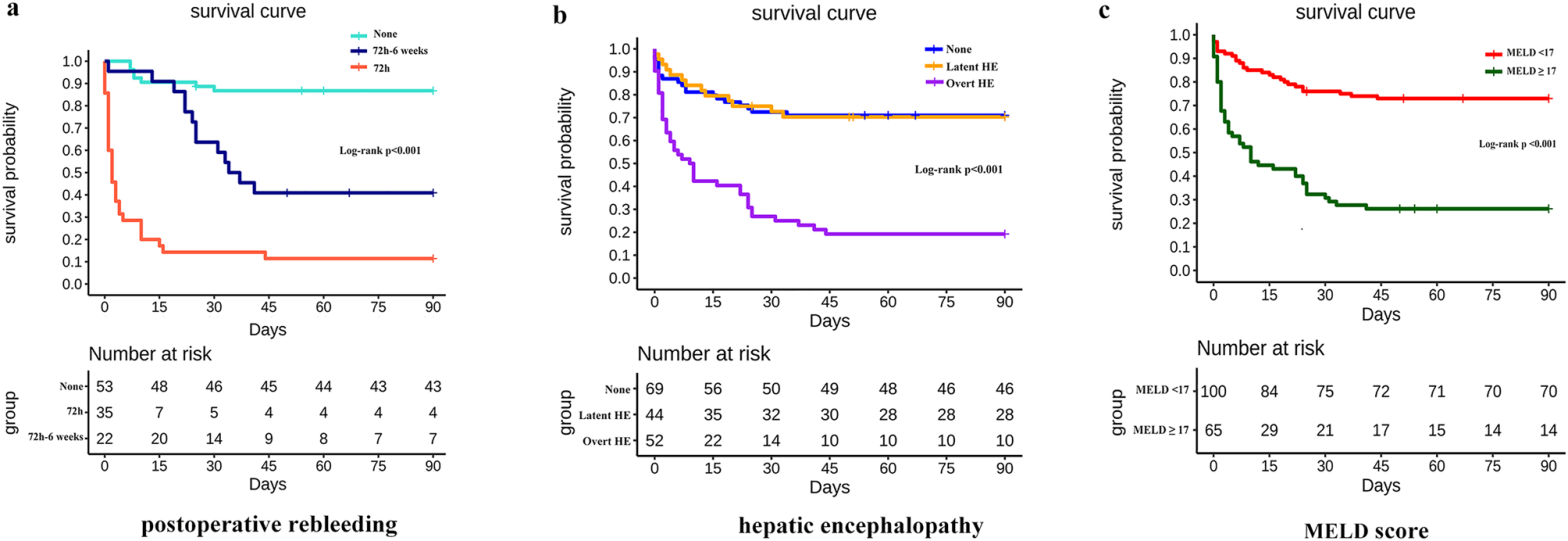
Kaplan–Meier analysis of three months survival following the independent risk factors of 6-week mortality. (**a**) postoperative Rebleeding; (**b**) Hepatic encephalopathy; (**c**) MELD score.

According to our risk factors analysis of 6-week mortality, postoperative rebleeding, including failure to achieve hemostasis and early rebleeding, was the main cause of death within 6 weeks. We further analyzed the cumulative three months survival rates by the Kaplan–Meier method. The results showed that the postoperative survival of patients with treatment failure and early rebleeding was shorter than that of patients with treatment success and these without early rebleeding (Fig. 4a). Overt hepatic encephalopathy (Fig. 4b) and a high MELD score (Fig. 4c) were also positively associated with the risk of death.

## Discussion

Acute EGVB represents a severe medical emergency characterized by a high mortality rate. To ensure patient safety and improve prognosis, emergency endoscopic hemostasis under intubation anesthesia in the ICU is considered to be essential and typically performed under intubation anesthesia within 12–24 h after the onset of bleeding^15^. However, this procedure is often performed during the active bleeding phase, resulting in a restricted visual field, which may increase the risk of hemostasis failure, recurrent bleeding in EGVB patients^8^. Although prognostic models are commonly used to assess the severity risk of liver disease and to stratify the risk of gastrointestinal bleeding^16^, there are a few research on the specific risk factors associated with emergency endoscopic treatment under tracheal-intubation supervision in patients with acute severe EGVB. This study aimed to develop and validate nomograms prediction models related to the risk factors for hemostasis failure and 6-week mortality, which can provide important clinical guidance for subsequent management of severe EGVB. In results, higher WBC counts, CTP scores, and the presence of shock were independently associated with hemostasis failure. A predictive nomogram model incorporating these factors showed good accuracy in assessing the risk of failure. Furthermore, another predictive model for 6-week mortality highlighted the significance of MELD score, hepatic encephalopathy, and postoperative rebleeding.

The primary objectives of emergency endoscopic therapy in patients with acute EGVB are to prevent recurrent bleeding and reduce mortality. Previous studies have shown that the prognosis of endoscopic treatment in patients with EGVB was closely related to the severity of liver disease, bacterial infection, higher hepatic venous pressure gradient (HVPG) levels, and hypovolemic shock^17,18^. In this study, we evaluated the efficacy and risk factors of bedside emergency endoscopic treatment with endotracheal intubation protection in acute EGVB patients presenting with shock, actively vomiting blood, agitation, altered consciousness such as latent hepatic encephalopathy. The findings revealed a higher overall rate of endoscopic hemostasis failure (32.1%, 53/165) compared to the existing literature^19,20^, which might be attributed to the challenges posed by more advanced and complex in liver disease severity in acute EGVB patients in this study and a compromised endoscopic visual field during emergency treatment, making hemostasis more difficult. Although erythromycin administration prior to endoscopy has been shown to improve the visualization of the mucosa and outcomes for GI bleeding, erythromycin cannot be applied in our study because its inaccessibility. Furthermore, higher WBC counts and CTP scores were independent risk factors for hemostasis failure. but also incorporates clinical manifestations (especially ascites) related to portal hypertension that are directly associated with EGVB due to a variety of causes. CTP score was initially designed to reflect the severity of liver disease, which not only considers liver function (bilirubin, albumin, prothrombin time) but also incorporates clinical manifestations. A higher CTP score in liver EGVB patients undergoing endoscopic treatments is likely to indicate poor clinical outcomes^21–23^, including hemostasis failure and death. The results of a cross-sectional study have in EGVB patients have shown that the CTP score upon the time of referral of a patient with varicose hemorrhage provides valuable insights into the risk of bleeding. Particularly, class B CTP was strongly susceptible to re-bleeding^23^. Moreover, cirrhosis severity is the main independent predictive factor for the development of infection after bleeding^17,24^. Compared with CTP score, MELD score mainly focuses on laboratory indicators (bilirubin, international normalized ratio and creatinine), which reflect liver function and extrahepatic failure, which is mainly used in the short- and medium-term mortality of end-stage liver disease. The results of this study showed that the WBC counts were higher in the failed hemostasis group than in the successful hemostasis group, which was not parallel to the reduction of Hb and PLT, and may be related to the presence of infections. Thus, this finding provides evidence to support the early use of antibiotic prophylaxis to preventing infections from admission^25^.

Comorbid shock is another independent predictive factor for endoscopic hemostasis failure. Patients with acute massive EGVB are prone to hemodynamic instability and altered consciousness. Therefore, volume restitution should be initiated to restore and maintain hemodynamic stability. Kim et al. conducted a retrospective study of 454 EVB patients with emergency EVL treatment and found that initial hypovolemic shock, and active bleeding on endoscopy were risk factors for EVL failure^8^. Similarly, Liu et al. reported that in patients classified as CTP class C with active bleeding on endoscopy, and the presence of PVT were associated with emergency EVL treatment failure. In together, when patients with acute massive EGVB exhibit significantly decreased hemoglobin, high CTP score and WBC counts, it is imperative to promptly assess these risk factors and initiate active intervention with symptomatic measures to increase the success rate of hemostasis under emergency gastroscopy. Finally, patients with failed hemostasis should be treated with prompt surgical interventions, such as liver transplantation, or trans-jugular intrahepatic portosystemic shunting.

Although endoscopic treatment can prevent or effectively control EGVB, portal hypertension cannot be fundamentally eliminated, and patients may experience rebleeding even after successful endoscopic hemostasis^26^. According to the natural course of EGVB, rebleeding within 6 weeks is regarded as early rebleeding^27^. Previous reports showed that early rebleeding occurred in more than 20–40% of EGVB patients which was associated with the poor prognosis^28,29^. In our study, we observed an overall early rebleeding rate of 29.3%, which was significantly associated with high CTP score (OR 1.50, 95% CI 1.15–1.94; *p* = 0.003) and gastric varices (OR 5.85, 95% CI 1.55, 22.05; *p* = 0.009), which were consistent with previous reports^30^. Gastric variceal bleeding is typically more difficult to control than esophageal variceal bleeding, however, its occurrence is less common, accounting for nearly 20% of all EGVB cases^31^. Recently, ETA has been regarded as the preferred treatment modality for gastric variceal with significant hemostatic effects. However, tissue glue, as a foreign matter, starts to seep into the gastric cavity 1 month after hemostatic treatment, which may result in bleeding during the gel extrusion and the probability of early re-emergence. A previous study reported that gastric variceal had a high rebleeding rate of 34–89% during glue extrusion period^32^. Apart from these, gastric varices, encephalopathy (meaningful in univariate analysis and not meaningful in multifactorial logistic analysis in our study), portal thrombosis, HCC, large varices, active bleeding during endoscopy, and a high HVPG levels have been found to increase the risk of in early rebleeding^33,34^.

6-week mortality is currently considered the primary prognostic endpoint for assessing the efficacy of emergency endoscopic hemostatic therapy in patients with acute EGVB^4,5^. It has been found to be related to CTP score, MELD score, AIMS65 score, and comorbidities^35^. In our study, the mortality rate within 6 weeks was 45.45% (75/165), which was higher than those reported previously^36^. This observation may be attributed to the patients who died having a more advanced in liver disease severity, as evidenced by higher MELD and CTP score. Additionally, these patients with acute EGVB often presented with multiple comorbidities, including shock, PVT, hepatic encephalopathy, and ascites. The superiority of the MELD model in comparison to the CTP score in determining 6-week mortality after acute EGVB has been demonstrated^37,38^. Consistently, a high MELD score rather than CTP score was considered as an independent risk factor in our study. Notably, overt hepatic encephalopathy, an important component of the CTP score, was also found to be related to 6-week mortality. It is well-acknowledged that hepatic encephalopathy can be precipitated by EGVB. Overt hepatic encephalopathy is defined as a condition with significant clinical manifestations such as disorientation or fluttering tremor episodes with highly mortality estimated to be 40% at 1 year^39^. Accordingly, in our study, we found that overt hepatic encephalopathy was related to 6-week mortality in EGVB patients. Moreover, postoperative rebleeding was another important independent risk factor for 6-week mortality. The survival of patients with postoperative rebleeding was shorter than that of patients without rebleeding. The nomogram containing the MELD score, hepatic encephalopathy, and postoperative rebleeding showed a strong predictive ability for 6-week mortality in patients with EGVB who underwent bedside emergency endoscopic treatment with endotracheal intubation protection.

To the best of our knowledge, this study is the first attempt to develop predictive nomograms based on patients with severe EGVB who underwent bedside emergency endoscopic treatment following endotracheal intubation protection. However, this study had a few limitations. First, this was a small-scale single-center retrospective study, that undoubtedly had a degree of selection bias and confounding factors. Second, patients with acute severe EGVB patients required to receive emergency endoscopic treatment quickly and it is difficult to refine the associated imaging and hepatic venous pressure gradient in the acute setting. Therefore, our study missed several important imaging data such as liver stiffness and spleen length. Large multicenter and prospective studies are required for further verification and investigation.

## Conclusion

In conclusion, CTP score, WBC counts and hemorrhagic shock were selected to predict the risk of hemostasis failure in bedside emergency endoscopy treatment following endotracheal intubation. Moreover, the 6-week mortality was found to be associated with recurrent bleeding, high MELD score and overt hepatic encephalopathy. Accordingly, we respectively established two nomogram models with good predictive accuracy to evaluate the risk of hemostasis failure and 6-week mortality in patients with severe EGVB who underwent bedside emergency endoscopic treatment under endotracheal intubation protection. The risk stratification regarding the endoscopic emergency hemostasis failure rate and early rebleeding is of great significance for guiding clinical decisions to improve the prognosis of severe EGVB in ICU. If the failure rate of emergency endoscopic treatment following endotracheal intubation is assessed to be high, early salvage treatment (72 h) should be considered in advance.

### Supplementary Information

Supplementary Table S1.

## Data Availability

The datasets used in this study are available on reasonable request to the corresponding author.
