# Supplementary material for: Risk factors and predictive nomograms for bedside emergency endoscopic treatment following endotracheal intubation in cirrhotic patients with esophagogastric variceal bleeding
Source: Sci Rep. 2024 Apr 24;14:9467. doi: 10.1038/s41598-024-59802-0 (PMC11043454; doi:10.1038/s41598-024-59802-0)
Supplement: Supplementary file 1 — Supplementary Table S1. [file 41598_2024_59802_MOESM1_ESM.pdf]

**Supplementary Table**

**Table S1.** The risk factors associated with the early rebleeding in EGVB patients in training cohort

| Variables | Early rebleeding | | Univariate | | Multivariate | |
|---|---|---|---|---|---|---|
| | Yes (n=22) | No (n=53) | OR (95% CI) | p value | OR (95% CI) | p value |
| Age | 58 ± 2.8 | 55.5 ± 1.7 | 1 (0.4-2.6) | 0.446 | | |
| WBC ($10^9$/L) | 7.9 ± 1.1 | 7.5 ± 0.7 | 0.9 (0.4-1.9) | 0.767 | | |
| Hb (g/L) | 65.1 ± 4.2 | 59.8 ± 2.3 | 1.1 (0.4-2.7) | 0.230 | | |
| PLT ($10^9$/L) | 89 ± 14.6 | 80.7 ± 6.3 | 0.7 (0.2-1.7) | 0.547 | | |
| ALT (U/L) | 27 (17.5-43.3) | 22 (11-46.5) | 1.8 (0.7-5) | 0.361 | | |
| AST (U/L) | 37 (26.3, 133) | 38 (20, 93) | 0.7 (0.3-1.9) | 0.442 | | |
| TBIL (µmol/L) | 48.2 (17.7-135) | 30.7 (18.4-69) | 1.3 (0.5-3.5) | 0.346 | | |
| ALB (g/L) | 27.7 ± 1.3 | 26.9 ± 0.8 | 0.9 (0.3-2.3) | 0.591 | | |
| CHE (µmol/L) | 2284 ± 209 | 2243 ± 151 | 1.1(0.6-1.8) | 0.886 | | |
| PT (s) | 17.7 (13.9-27.3) | 19 (15.1-24.3) | 1 (0.6-1.7) | 0.944 | | |
| PTA (%) | 50.9 ± 4.8 | 46.4 ± 2.2 | 1.3 (0.5-3.4) | 0.391 | | |
| INR | 1.71 (1.2-2.5) | 1.7 (1.4-2.1) | 1.2 (0.7-1.9) | 0.745 | | |
| CTP scores | 11.5 ± 0.6 | 10.1 ± 0.3 | 3.3(1.2-9.4) | 0.023 | 1.5 (1.2 - 1.9) | 0.003 |
| MELD scores | 18.1 ± 2.4 | 15.4 ± 1.2 | 1.1(0.4-3) | 0.257 | | |
| Sex, male | 18 (81.8) | 37 (69.8) | 2 (0.6-6.7) | 0.284 | | |
| Shock | 14 (63.6) | 25 (47.2) | 0.5 (0.2-1.4) | 0.194 | | |
| First bleeding | 11 (50) | 20 (37.7) | 0.6 (0.2-1.7) | 0.326 | | |
| Portal vein emboli | 10 (45.5) | 22 (41.5) | 0.9 (0.3-2.3) | 0.753 | | |
| Liver cancer | 8 (36.4) | 11 (20.8) | 0.5 (0.2-1.4) | 0.157 | | |
| Hepatic failure | 6 (27.3) | 17 (32.1) | 1.3 (0.4-3.8) | 0.681 | | |
| Bleeding site, EV | 13 (59.1) | 43 (81.1) | 3 (1.1 - 8.9) | 0.046 | 5.9 (1.6, 22.1) | 0.009 |
| Blakemore tube | 10 (45.5) | 21 (39.6) | 0.8 (0.3-2.2) | 0.641 | | |
| Treatment | | | 1.6 (0.6-4.4) | 0.347 | | |
| TAI/EIS/EVL | 9 (41.7) | 28 (52.8) | | | | |
| TAI+EIS/EVL | 13 (58.3) | 25 (47.2) | | | | |
| HE | | | | 0.026 | | 0.265 |
| No | 6 (27.3) | 31 (58.5) | Reference | | | |
| Latent | 8 (36.35) | 15 (28.3) | 2.8 (0.8-9.4) | 0.105 | 2.1 (0.6-8) | 0.283 |
| Overt | 8 (36.35) | 7 (13.2) | 3.1 (1.4-7) | | 3.7 (0.7-19.8) | 0.133 |
| Ascites | | | | 0.112 | | |
| No | 0 (0) | 6 (11.3) | Reference | | | |
| Mild | 8 (36.4) | 25 (47.2) | 4.3 (0.2-86.3) | 0.335 | | |
| Moderate-severe | 14 (63.6) | 22 (41.5) | 8.4 (0.4-160.3) | 0.158 | | |
